# Metabolic Profiling Reveals Distinct Variations Linked to Nicotine Consumption in Humans — First Results from the KORA Study

**DOI:** 10.1371/journal.pone.0003863

**Published:** 2008-12-05

**Authors:** Rui Wang-Sattler, Yao Yu, Kirstin Mittelstrass, Eva Lattka, Elisabeth Altmaier, Christian Gieger, Karl H. Ladwig, Norbert Dahmen, Klaus M. Weinberger, Pei Hao, Lei Liu, Yixue Li, H.-Erich Wichmann, Jerzy Adamski, Karsten Suhre, Thomas Illig

**Affiliations:** 1 Institute of Epidemiology, Helmholtz Zentrum München, German Research Center for Environmental Health, Neuherberg, Germany; 2 Bioinformatics Center, Key Lab of Systems Biology, Shanghai Institutes for Biological Sciences, Chinese Academy of Sciences, Shanghai, People's Republic of China; 3 Institute of Bioinformatics and Systems Biology, Helmholtz Zentrum München, German Research Center for Environmental Health, Neuherberg, Germany; 4 Institute of Medical Informatics, Biometry and Epidemiology, Ludwig-Maximilians-Universität, Munich, Germany; 5 Department for Psychiatry, University of Mainz, Mainz, Germany; 6 Biocrates Life Sciences AG, Innsbruck, Austria; 7 Shanghai Center for Bioinformation Technology, Shanghai, People's Republic of China; 8 Institute of Experimental Genetics, Genome Analysis Center, Helmholtz Zentrum München, German Research Center for Environmental Health, Neuherberg, Germany; 9 Lehrstuhl für Experimentelle Genetik, Technische Universität München, Munich, Germany; 10 Faculty of Biology, Ludwig-Maximilians-Universität, Planegg-Martinsried, Germany; Yale University School of Medicine, United States of America

## Abstract

Exposure to nicotine during smoking causes a multitude of metabolic changes that are poorly understood. We quantified and analyzed 198 metabolites in 283 serum samples from the human cohort KORA (Cooperative Health Research in the Region of Augsburg). Multivariate analysis of metabolic profiles revealed that the group of smokers could be clearly differentiated from the groups of former smokers and non-smokers. Moreover, 23 lipid metabolites were identified as nicotine-dependent biomarkers. The levels of these biomarkers are all up-regulated in smokers compared to those in former and non-smokers, except for three acyl-alkyl-phosphatidylcholines (e.g. plasmalogens). Consistently significant results were further found for the ratios of plasmalogens to diacyl-phosphatidylcolines, which are reduced in smokers and regulated by the enzyme alkylglycerone phosphate synthase (alkyl-DHAP) in both ether lipid and glycerophospholipid pathways. Notably, our metabolite profiles are consistent with the strong down-regulation of the gene for alkyl-DHAP (*AGPS*) in smokers that has been found in a study analyzing gene expression in human lung tissues. Our data suggest that smoking is associated with plasmalogen-deficiency disorders, caused by reduced or lack of activity of the peroxisomal enzyme alkyl-DHAP. Our findings provide new insight into the pathophysiology of smoking addiction. Activation of the enzyme alkyl-DHAP by small molecules may provide novel routes for therapy.

## Introduction

An estimated one billion men and 250 million women worldwide are daily tobacco smokers, primarily through cigarettes [1]. Cigarette smoking is the cause of about 90 percent of the world's lung cancer cases, and accounts for one in four cancer deaths worldwide [2], [3], [4]. Smoking decreases high density lipoprotein (HDL) carrying cholesterol, thus increasing the risk for many cardiovascular diseases. The incidence of acute myocardial infarction is about 2.5 times higher in smokers than in non-smokers, according to a study grounded on the population-based research platform KORA (Cooperative Health Research in the Region of Augsburg) [5], [6], [7].

Metabolites are the intermediate or end points of metabolism, and biomarkers refer to indicators of a particular disease state or a particular physiological state of an organism. In cigarette smoke, there are more than 5,000 chemicals, including about 70 cancer-causing agents (carcinogens), among which nicotine and its major metabolite cotinine and carbon monoxide are found to be biomarkers of cardiovascular damage [8], [9]. After cigarette smoke is inhaled, nicotine is carried deep into the lungs, where it is absorbed into the bloodstream and carried to almost every part of the body. Nicotine reaches the brain within 10 seconds, and has been found in breast milk as well as in the umbilical blood of newborn babies.

There have been a few studies addressing metabolite changes in smokers. Several metabolites, including carbon monoxide, metabolites of the tobacco-specific carcinogen 4-(methylnitrosamino)-1-(3-pyridyl)-1-butanone (NNK), and total cotinine (cotinine plus cotinine-N-glucuronide), were investigated in urine samples of a study in which the number of cigarettes consumed was reduced daily. No significant differences were observed, presumably due to a potential compensation mechanism [10], [11]. It was suggested that people who are trying to cut back by consuming fewer cigarettes per day change their behavior by inhaling longer and deeper, which is known to alter a smoker's exposure to carcinogens.

Due to a lack of powerful tools for analyses, large-scale metabolic screens of smoking phenotype in blood plasma or serum have not been reported to date. In recent years, technology improvements have greatly advanced the field of metabolomics, which involves rapid, high-throughput characterization of the small molecular metabolites identified in an organism [12], [13], [14]. Metabolite profiles are very much dependent on the genetic background and the physiological status of the organism. They are also dependent on environmental factors and are regarded as the ultimate result of cellular regulation, resulting in the observed phenotypes [15], [16], [17], [18]. However, the classes and numbers of detected metabolites are still limited to date.

In the current study, we investigated concentrations of 198 metabolites in 283 KORA samples by targeted metabolomics, in order to study the influence of cigarette smoking on blood serum profiles. We systematically analyzed the metabolic profiles employing several statistical methods, such as simple calculation of the correlations of metabolites and the ratios of metabolite concentration, metabolites clustering, multivariate statistics (Partial Least Squares Discriminant Analysis, PLS-DA, Principal Component Analysis, PCA and Correspondence Analysis, CA) [14], [19], [20], [21], [22], as well as ANOVA [23] and Wilcoxon tests [24], [25]. We investigated the populations at individual and group levels and observed significant changes of two types of metabolites, which are intermediates or end products of glycerophospholipid- and ether lipid-metabolism, in smokers compared to former and non-smokers. Based on our own data and as well on another gene expression study, we propose a molecular mechanism explaining the altered lipid balance in smokers.

## Results

### Clustering of human metabolites in the KORA population

**Figure 1:**
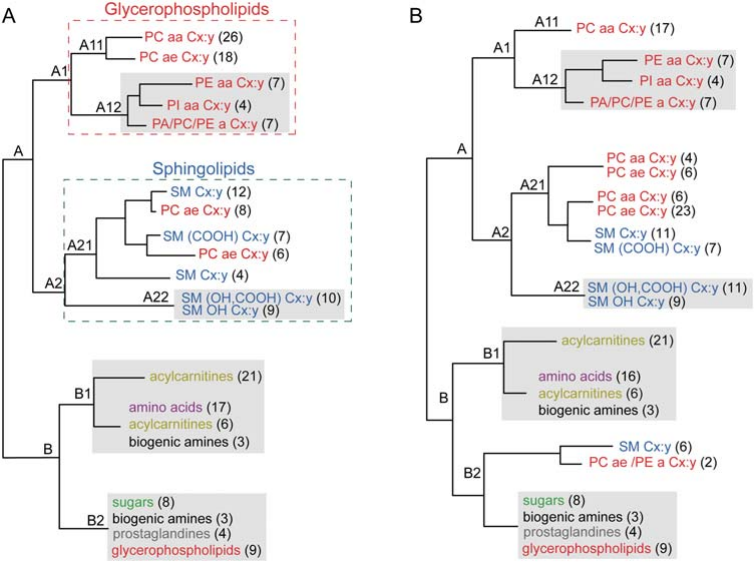
Comparison of clustering results. (A) Classification of the 198 metabolites based on population-based KORA samples (*n* = 283). Similar classes of metabolites are shown with the same color. The numbers in brackets next to the metabolite name indicate how many metabolites are included. In some clusters, one other class of metabolite was clustered together, did not indicated here. (B) Classification of the 198 metabolites by removing the 28 current smokers from the date set (*n* = 255). Similar clusters in plots A and B are shown with gray shadows. For more details, see Figures S1 to S4.

In total, 283 human blood sera were analyzed and 198 metabolites were obtained for each individual (see Materials and Methods). An correlation matrix of all metabolite concentrations was calculated based on the 283 individuals and hierarchical clustering resulted in two main clusters, A and B (Figures 1A, S1 and S2). Cluster A consists of lipids and has two sub clusters: glycerophospholipids (cluster A1) and sphingolipids (cluster A2), except for 14 acyl-alkyl- (*ae*) phosphatidylcholines. For classes and biochemical names of the metabolites see Table S1. In general, metabolites with similar polar head groups and the same type of side chains were found to be closely clustered. For example, sub cluster A11 consists of the same head group phosphatidylcholines (PC), and is differentiated by those with diacyl- (*aa*) vs. *ae*-phosphatidylcholines (Figure 1A); three sub clusters were obtained in A12, phosphatidylethanolamines (PE) and phosphatidylinositols (PI) with *aa* bonds, the third sub cluster comprises lipids with one side chain, but three head groups (PC, PE and PA for phosphatidic acids). Cluster B also consists of two sub clusters–acylcarnitines together with amino acids (cluster B1) and biogenic amines (cluster B1 and B2), and prostaglandins with sugars (cluster B2), except for nine glycerophospholipids. Related classes of metabolites are generally clustered together based on the population-based KORA samples.

When the influence of smoking on the human metabolome was investigated, 28 current cigarette smokers were removed from the sample set to ensure statistical accuracy. A slightly different clustering of metabolites was consequently observed (Figure 1B and Figures S3, S4). Overall, cluster B did not change, except that six sphingomyelins and two glycerophospholipids moved from cluster A to B2. A decreased level of correlation of the glycerophospholipids was seen in cluster A (Figures S1, S2, S3 and S4). Furthermore, some glycerophospholipids were found to be closely related with sphingolipids in clusters A21. This suggested that some lipids were affected because of the removal from the dataset of the data for the 28 smokers.

To investigate the significance of observed differences between the full dataset and that with the removal of the 28 current smokers, 200 permutations were conducted by randomly sampling 255 individuals (i.e. removing 28 samples without replacement) from the “283 dataset”, and a correlation matrix of all metabolites was calculated. The resulting 198×198 matrix was correlated with the one obtained using the original “283 dataset”. Each of the two matrices was first converted into a vector. The Pearson's correlation coefficient of these vectors was then calculated. The normal distribution of these 200 coefficients has been used in a *t-*test as a null hypothesis. It is significantly different from the one in which the 28 smokers were removed (*p*-value of *t-*test was 2.28E-4) and correlated with the pair-wise matrices of the original 283 dataset, suggesting that the observed differences after the removal of the 28 smokers are not a random effect.

### Metabolic profiles differentiate current smokers from former and non-smokers

**Figure 2:**
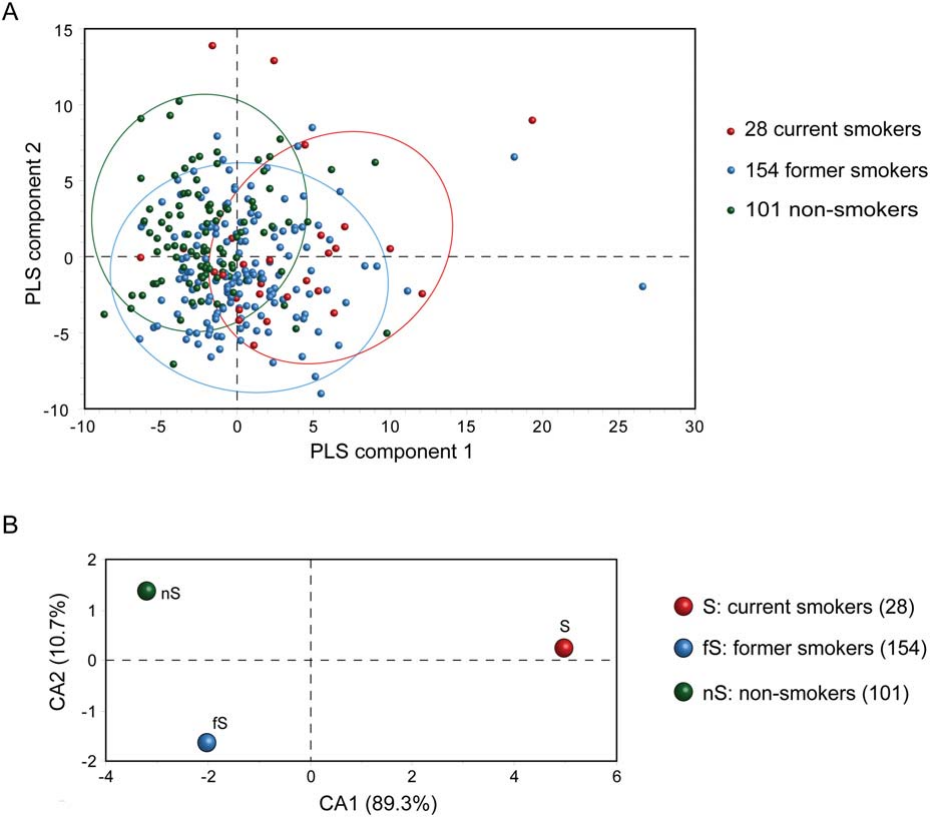
Multivariate analysis results. (A) Two dimensional PLS-DA results of 283 individuals. The 28 current smokers are displayed in red, while 154 former smokers and 101 non-smokers are indicated in blue and green, respectively. Three dimensional PLS-DA results are shown in Figure S5A. (B) CA results of the current, former and non-smoker groups.

When Partial Least Squares Discriminant Analysis (PLS-DA), Principal Component Analysis, (PCA) and Correspondence Analysis (CA) were applied for the 283 individuals with 198 metabolites, current, former and non-smokers could be separated to a certain extent (Figure 2A, PCA and CA results are shown in Figure S5). When the three groups based on the mean value of the metabolites were characterized, CA results showed that smokers separated clearly from former and non-smokers by the first CA component, which accounted for 89 percent of the total variance (Figure 2B).

The first component is dominated by a set of metabolites (Table 1), indicating that these metabolites are primarily responsible for separating smokers from former and non-smokers. The higher the CA score, the more it contributes to the separation. It is the second CA component, which accounts for 11 percent of the total variance of CA that distinguishes non-smokers from former smokers in the dataset. The second component is dominated by two sphingolipids with high CA2 scores (Table 1), suggesting these two metabolites are sensitive for distinguishing former smokers from non-smokers.

**Table 1:** Identified nicotine-dependent potential biomarkers

Metabolite	Mean±Standard Error (µM)			CA scores		ANOVA	Wilcoxon (*p*-value)	
	Smoker	Former smoker	Non-smoker	CA1	CA2	*p*-value	S-nS	fS-nS
PC aa C32:1	71.09±6.01	52.77±1.90	45.52±1.55	1.13	0.17	6.9E-07	1.5E-06	0.039
PC aa (OH, COOH) C30:3	241.3±17.7	192.1±4.53	174.4±4.07	1.41	0.17	4.1E-07	4.4E-05	0.024
PC aa C34:1	530.4±38.8	417.5±10.2	389.3±9.26	2.06	0.04	2.0E-06	3.6E-05	0.22
PC aa C34:0	72.37±4.37	57.57±1.61	52.2±1.43	0.78	0.10	3.6E-06	7.2E-06	0.079
PC aa C36:1	172.6±11.7	139.4±3.34	133.6±3.07	0.93	−0.07	4.9E-05	1.9E-04	0.34
PC aa C36:2	490.3±33.2	402.6±8.56	406.8±9.12	1.08	−0.48	5.4E-04	2.3E-02	0.49
PC aa C36:3	299.8±24.5	245.7±4.95	244.17±6.38	0.94	−0.28	7.7E-04	5.7E-03	0.72
PC aa C38:2	70.10±3.54	58.21±1.16	58.73±1.34	0.37	−0.17	3.6E-04	2.7E-03	0.69
PC aa C38:3	152.6±9.80	121.8±3.04	116.6±3.42	0.94	−0.07	6.8E-05	1.4E-04	0.26
PC aa C40:4	12.59±0.85	9.57±0.45	8.63±0.29	0.40	0.03	5.4E-04	4.3E-06	0.21
PC aa C40:5	35.40±2.42	27.58±1.02	25.26±0.70	0.58	0.04	1.2E-04	7.6E-05	0.34
PC aa C32:0	33.04±1.99	28.59±0.71	26.53±0.66	0.28	0.06	1.2E-03	1.8E-03	0.21
PE aa C34:2	3.24±0.44	2.20±0.10	1.98±0.12	0.29	0.01	1.7E-04	2.8E-03	0.083
PE aa C36:2	6.55±0.95	4.40±0.21	3.88±0.22	0.43	0.02	6.9E-05	7.0E-04	0.14
PE aa C36:4	3.80±0.41	2.73±0.12	2.44±0.16	0.27	0.02	4.3E-04	8.0E-05	0.015
PE aa C38:4	7.47±0.82	5.38±0.27	4.75±0.27	0.38	0.03	5.9E-04	4.1E-04	0.056
PI aa C36:4	3.84±0.35	2.93±0.12	2.64±0.13	0.22	0.02	1.0E-03	2.0E-03	0.18
PI aa C38:4	33.39±2.58	26.92±0.93	25.46±0.85	0.43	0.01	3.0E-03	1.4E-03	0.37
PC ae C40:6	7.90±0.69	8.78±0.21	8.78±0.21	−0.33	0.10	1.4E-02	5.3E-04	0.024
PC ae C36:2	25.03±1.73	24.62±0.53	26.57 ±0.60	−0.28	0.19	7.3E-02	0.037	0.011
PC ae C38:2	12.61±0.78	12.42±0.24	13.69±0.33	−0.22	0.16	8.2E-03	0.059	4.5E-03
SM (OH, COOH) C16:1	33.89±2.41	28.38±0.77	23.64±0.91	0.53	0.24	1.1E-06	3.4E-05	1.6E-05
SM OH C20:3	195.9±9.81	179.1±4.48	157.1±4.74	0.54	0.47	2.9E-04	2.3E-04	1.2E-03

Metabolite names are listed in the first column (for classes and biochemical names, see Table S1). Mean values with standard error of metabolite concentrations in µM for each group, current smoker (S), former smoker (fS) and non-smoker (nS), are shown in columns 2 to 4, respectively. The first and second component score of the CA are given in columns 5 and 6. In the last three columns, *p*-values of ANOVA and Wilcoxon tests are indicated.

### Novel nicotine-dependent biomarkers

Potential nicotine-dependent (ND) biomarkers were identified using various statistical methods (Table 1). For example, for metabolite PC aa C32:1, the mean values of the current smoker (S), former smoker (fS) and non-smoker (nS) were 71.09, 52.77 and 45.52 µM, respectively; ANOVA tests of these mean values and the results showed that these differences are highly significant (*p*-value 6.9E-07). Wilcoxon tests of the differences between 28 S and 101 nS also indicated high significance with (*p*-value 1.5E-06); Wilcoxon tests for the differences between 154 fS and 101 nS are also significant at the 5% level. In addition to PC aa C32:1, seven metabolites differing between fS and nS were found to be significant at 5% level based on the Wilcoxon test (Table 1). Especially two sphingomyelins, SM (OH, COOH) C16:1 and SM OH C2:3, and one PC ae C38:2 had the most significant *p*-values in the Wilcoxon test comparing former smokers and non-smokers. These two sphingomyelins were also identified by CA method (i.e. have high CA2 scores, see above).

**Figure 3:**
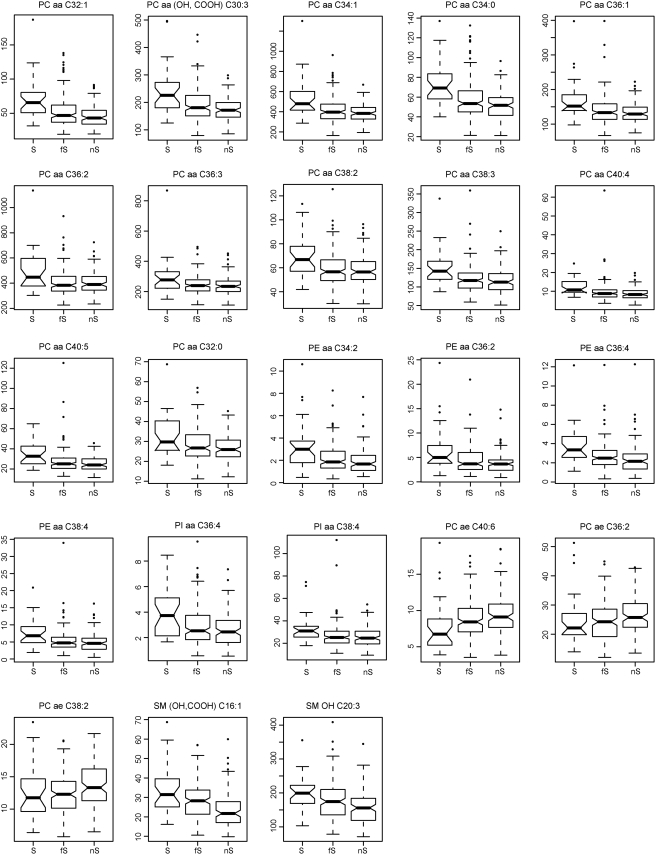
Box plots of the 23 identified nicotine-dependent potential biomarkers. For each metabolite, box plot of current (S), former (fS) and non-smoker (nS) groups is illustrated. For each group, the five parameters are the smallest concentration of the metabolite, lower quartile, median, upper quartile, and largest observation. The points outside the quartiles are outliers.

For the 23 potential ND biomarkers, the mean values of current, former and non-smoker groups are clearly distinct (Table 1). Differences were observed based on the median value of the three groups, with a few outliers for each metabolite (see box plots in Figure 3). The biomarker levels in current smokers are almost all up regulated compared to those in former and non-smokers, with three acyl-alkyl-phosphatidylcholines (PC ae C40:6, PC ae C36:2 and PC ae C38:2) down regulated.

### Ratios of acyl-alkyl- to diacyl-phosphatidylcholines are reduced in smokers compared to non-smokers

**Figure 4:**
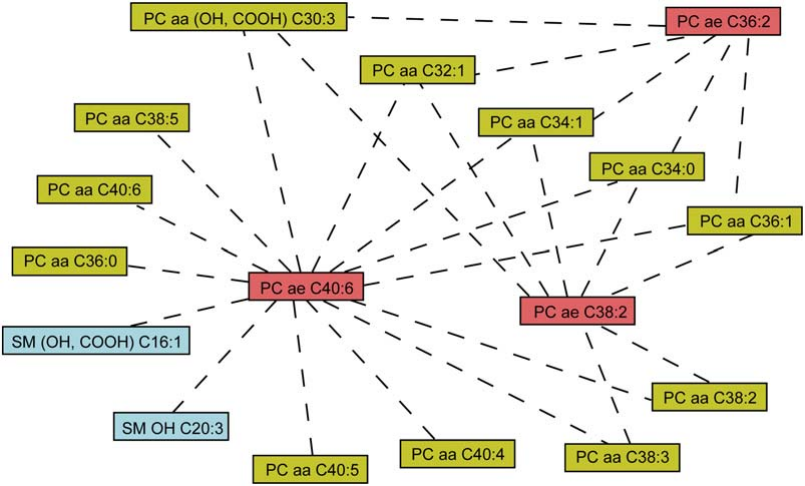
Significantly corrected pairs of metabolites with smoking phenotype. Significantly correlated pairs of metabolites are demonstrated by dashed lines. For more details, see Table 2. Similar classes of metabolites are shown with the same color.

To further investigate the observed three plasmalogens deficiency, we calculated ratios of all pair metabolite concentrations and correlated them with nicotine consumption (see Materials and Methods). The most significantly correlated pairs of metabolites are listed in Table 2 and are illustrated in Figure 4. For example, with the smoker phenotype, ratio PC ae C40:6/PC aa C32:1 is positively significantly correlated (*r* is 0.333, and *p*-value of *t*-test is 9.5E-09), while ratio PC aa C32:1/PC ae C40:6 is negatively significantly correlated (*r* is −0.378 with *p*-value 5.0E-11). These data indicate that in smokers, the relative concentration of PC ae C40:6 was significantly lower than PC aa C32:1, which is inconsistent with the observation in single metabolite analysis (Table 1). Moreover, the ratios of metabolite PC ae C40:6 with other 13 metabolites were all significant, suggesting that plasmalogens (PC ae C40:6) are down regulated in smokers compared to other 11 diacylated phosphatidylcholines and two sphingomyelines. For the other two acyl-alkylated phosphatidylcholines, PC ae C38:2 and PC ae C36:2, there were seven and five significantly correlated diacyl-phosphatidylcholines, respectively.

**Table 2:** Significantly correlated ratios of metabolite concentration with smoker phenotype

Metabolite M1	Metabolite M2	*n*-cases	Ratio (M1/M2)		Ratio (M2/M1)	
			*r*	*p*-value	*r*	*p*-value
PC ae C40:6	PC aa C32:1	283	0.333	9.5E-09	−0.378	5.0E-11
PC ae C40:6	PC aa (OH, COOH) C30:3	283	0.317	4.8E-08	−0.398	3.7E-12
PC ae C40:6	PC aa C34:1	283	0.320	3.7E-08	−0.393	6.6E-12
PC ae C40:6	PC aa C34:0	283	0.339	4.8E-09	−0.393	7.1E-12
PC ae C40:6	PC aa C36:0	283	0.317	4.8E-08	−0.360	4.4E-10
PC ae C40:6	PC aa C38:5	283	0.317	5.3E-08	−0.346	2.3E-09
PC ae C40:6	PC aa C40:6	283	0.314	7.0E-08	−0.396	4.6E-12
PC ae C40:6	PC aa C38:3	283	0.317	5.1E-08	−0.395	5.6E-12
PC ae C40:6	PC aa C40:4	283	0.313	7.3E-08	−0.352	1.1E-09
PC ae C40:6	PC aa C40:5	283	0.356	7.0E-10	−0.403	1.9E-12
PC ae C40:6	PC aa C38:2	283	0.302	2.2E-07	−0.385	1.9E-11
PC ae C40:6	PC aa C36:1	283	0.305	1.6E-07	−0.387	1.5E-11
PC ae C40:6	SM (OH, COOH) C16:1	283	0.329	1.4E-08	−0.377	5.4E-11
PC ae C40:6	SM OH C20:3	283	0.315	6.5E-08	−0.345	2.4E-09
PC ae C38:2	PC aa C32:1	283	0.326	1.9E-08	−0.364	2.8E-10
PC ae C38:2	PC aa C38:3	283	0.313	7.9E-08	−0.346	2.2E-09
PC ae C38:2	PC aa C34:0	283	0.317	5.0E-08	−0.377	5.2E-11
PC ae C38:2	PC aa C36:1	283	0.316	5.5E-08	−0.358	5.7E-10
PC ae C38:2	PC aa C34:1	283	0.305	1.6E-07	−0.363	3.2E-10
PC ae C38:2	PC aa C38:2	283	0.300	2.8E-07	−0.342	3.6E-09
PC ae C38:2	PC aa (OH, COOH) C30:3	283	0.309	1.1E-07	−0.352	1.1E-09
PC ae C36:2	PC aa C36:1	283	0.320	3.7E-08	−0.335	7.3E-09
PC ae C36:2	PC aa (OH, COOH) C30:3	283	0.309	1.2E-07	−0.344	2.8E-09
PC ae C36:2	PC aa C34:0	283	0.304	1.8E-07	−0.342	3.7E-09
PC ae C36:2	PC aa C34:1	283	0.303	2.0E-07	−0.341	4.0E-09
PC ae C36:2	PC aa C32:1	283	0.301	2.5E-07	−0.327	1.7E-08

Two metabolites M1 and M2 are listed in the first two columns. Pearson's correlation coefficient *r* of metabolite ratios of M1/M2 and M2/M1 positively and negatively correlated with smoker phenotype are shown in columns 4 and 6, respectively. The corresponding *p*-values of *n*-cases of *t*-tests are indicated in columns 5 and 7, respectively.

For five metabolites, PC aa C32:1, PC aa (OH,COOH) C30:3, PC aa C34:1, PC aa C34:0 and PC aa C36:1, the ratios with the three acyl-alkyl-phosphatidylcholines were all significantly correlated with the nicotine consumption. These results further suggest that smokers have higher concentrations of diacyl-phospholipids and lower concentrations of the plasmalogens, whereas the opposite is seen in former and non-smokers. Notably, these five diacyl-phospholipids were found to have the most significant *p*-value of ANOVA and Wilcoxon tests based on the single metabolite study (Table 1). For the most statistically significant five metabolites, the results based on single metabolites and ratios of metabolites pairs agree with each other.

## Discussion

Our data provide clear evidence that metabolic profiling reflects human metabolism. We calculated correlation of all metabolite concentration pairs. Clustering results revealed that metabolites in related functional contexts are highly correlated. This is also consistent with similar conclusions of a mouse study based on 67 studied metabolites [26]. This demonstrates that metabolic profiles are biologically and statistically meaningful.

We applied our metabolic profiling to investigate the impact of cigarette smoking. Significant changes were observed mainly for clusters of lipid metabolites. Moreover, the 23 biomarkers that we could identify are all lipids, consistent with the observation that cell membranes are affected or damaged due to the influence of tobacco smoking [27]. The physiological importance of lipids is illustrated by the numerous diseases to which lipid abnormalities contribute, including atherosclerosis, diabetes, obesity, and Alzheimer's disease [28]. Lipids are major components of biological membranes, which maintain the integrity of cells and allow the compartmentalization of the cytoplasm into specific organelles. Cigarette smoke, then, might affect or even damage cell membranes, thus influencing the concentrations of related metabolites, namely the biomarkers discovered in this study.

**Figure 5:**
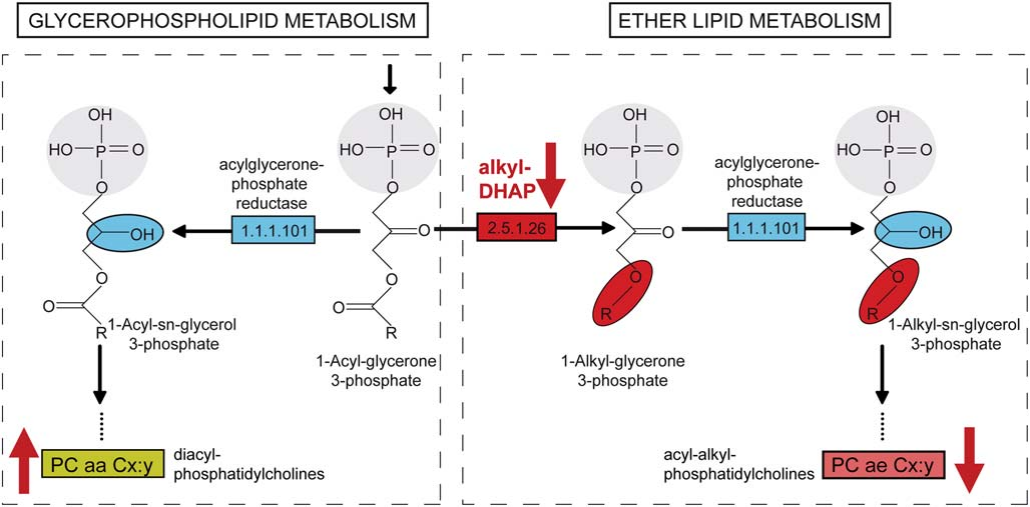
In smokers, reduced or lack of activity of the enzyme alkyl-DHAP may further regulate the ratio of acyl-alkyl- to diacyl- phosphatidylcholines in the ether lipid- and glycerophospholipid pathways. Part of the pathways of the glycerophospholipid- and ether lipid metabolism are shown. The names of the small molecular are indicated. Enzymes alkylglycerone phosphate synthase (alkyl-DHAP, EC 2.5.1.26) and acylglycerone phosphate reductase (EC 1.1.1.101) are shown in red and blue.

Glycerophospholipid metabolism and ether lipid metabolism share one small molecule, 1-acyl-glycerone 3-phosphate [29], [30], [31]. In the ether lipid metabolism pathway, a unique biochemical reaction is catalyzed by the enzyme alkylglycerone phosphate synthase (alkyl-DHAP, EC 2.5.1.26) resulting in the formation of the ether bond by replacement of the *sn*-1 fatty acid with a long chain fatty alcohol (Figure 5). The following biosynthesis steps in both ether lipid and diacyl-phospholipids converge in a reaction catalyzed by acylglycerone phosphate reductase (EC 1.1.1.101), which is utilized in the synthesis of both ether lipids and diacylated phopholipids [32]. Following similar synthesis steps in the ether lipid and glycerophospholipid pathways, acyl-alkyl-phosphatidylcholines and diacyl- phosphatidylcholines will be either intermediate or end products of the two pathways. Alkylglycerone phosphate synthase is encoded by *AGPS*, the gene in *Homo sapiens*. Interestingly, in a human lung project [33], it was found that the *AGPS* gene expression is highly increased in former and non-smokers relative to current smokers (Figure S6). The upregulation of alkyl-DHAP seen in our metabolite profiles and independent in a gene expression study further corroborates its role in defects linked to smoking.

Human newborns of nicotine-exposed pregnancies reveal growth retardation due to impairment of uteroplacental circulation as a result of the vasoconstricting effect of nicotine [34]. Studies in the rat showed that mechanisms involving deterioration development of fetal alveolae and up regulation of lipid peroxidation by P450 enzymes [35], [36]. In this respect, our study provides a novel insight in that nicotine affects plasmalogen levels. Plasmalogen comprise a major portion of the phospholipids in the adult human central nervous system. Overall, it was shown that newborn plasmalogen levels are relatively low (7% of total phospholipid mass) [32]. As the plasmalogens may influence the surface tension in alveolar surfactants [37], we hypothesize that this would be triggered as well by nicotine. Isolated (single gene defect) deficiency in human *AGPS* gene function further indicate that this gene is embryonic essential and its inactivation leads to a lethal phenotype [38], [39]. This gene is also affected in other disorders of biogenesis, such as Zellweger Syndrome or Rhizomelic chodrodisplasia punctata type 3 [40]. Therefore, all factors that influence ether lipid balance, including nicotine as shown here, are of potential risk to human health.

Our metabolic profiling provides a snapshot of the complex human metabolome. More detailed profiles in combination with kinetic experiments for blood sample collection are necessary to draw a comprehensive map and will reflect physiological processes as responses to developmental, genetic or environmental factors [16], [17], [41], [42].

The 198 detected metabolites are a large dataset in human blood samples, though much smaller in comparison to the human metabolomics database, which currently has a collection of about 2,500 metabolites [43]. Previously identified biomarkers of ND metabolites [9], [44], such as nicotine, cotinine and carbon monoxide, are not in our dataset. In addition to further technical improvements in metabolite detection sensitivity, samples from urine and other tissues are needed to enlarge our dataset.

Our study represents the first large screen of metabolites to study the influence of cigarette smoking on human blood serum. Albeit we are aware that the sample size of current smokers in this pilot study is small, our results are encouraging and we could show that the smokers are distinctly separated from former and non-smokers. In general, similar observations were obtained at an individual level, though with large variance. An interesting observation is that former smokers were found to be separated from non-smokers, suggesting that the influence of cigarette smoke in human blood remains for years. We note, however, that the group of former smokers is not well-defined in this study because the time when these individuals quit smoking is not documented. Damage to the cell membrane from smoking may be reversed over time due to the repair mechanisms in the human body [45], [46].

The independent but consistent observation from our metabolic profile analysis and *AGPS* gene expression data may indicate that smoking affects the enzymatic activity of alkyl-DHAP and thus change the ratios of two types of metabolites. However, the overall fat metabolism is likely not be affected, as the BMI does not vary significantly between the groups of current, former and non-smokers (data not shown).

Our analyses suggest that small molecules that activate the enzyme alkyl-DHAP could be developed to treat plasmalogens deficiency disorders that are caused by nicotine consumption in smokers.

## Materials and Methods

### Sample Source

KORA (Cooperative Health Research in the Region of Augsburg) is a population-based research platform with subsequent follow-up studies in the fields of epidemiology, health economics and health care research [5], [6], [7]. It is based on interviews in combination with medical and laboratory examinations, as well as the collection of biological samples. Answers from the participants were found to be reliable [47]. Details about the questionnaire forms and variables can be found at KORA-gen [5]: http://epi.gsf.de/kora-gen/. Four surveys were conducted with 18,079 participants. KORA-S3 consists of representative samples from 4,856 individuals. The dataset comprises individuals aged 25–74 years resident in the region of Augsburg, Southern Germany, examined in 1994–1995. During the years 2004–2005, 2,974 participants participated in a follow-up (KORA-F3) survey of the one conducted 10 years ago. For all studies, we obtained written consent from participants and approval from the local ethical committees.

### Sampling

Randomly selected population-based 283 male participants (aged 55–79 years) of KORA-F3 were used in the current study. Of the 283 individuals, 28 were current smokers (S), who smoked one to 50 (mean 17) cigarettes per day. Out of the 28 smokers, only nine completed the Fagerstöm test of ND form (FTND), the score of which reflects the addiction level of dependence on nicotine, and these data were not used in this study. Those who ceased smoking but smoked at least one cigarette daily were classified as former smokers. Non smokers had never smoked at the time when the study was conducted while 154 and 101 were former smokers (fS) and non-smokers (nS), respectively.

In KORA study, to characterize the nicotine consumption, the current smoker is defined as 1; sometimes smoker is defined as 2; former and non-smoker are quantified as 3 and 4, respectively.

Blood samples were collected in 2006. The standardized biological sample collections applied have been described in detail previously [5], [6], [7], [48]. Blood was drawn in the morning between 8 and 10 am and was immediately horizontal shaken for 10 minutes, followed by 40 minutes resting at 4°C to obtain complete coagulation, and finally centrifugation of blood was performed at 2000g, 4°C for 10 minutes for serum collection. Serum was aliquoted and kept for 2–4 hours at 4°C, after which it was frozen at −80°C until metabolic analyses.

### Metabolite measurements

Targeted metabolite profiling by electrospray ionization (ESI) tandem mass spectrometry (MS/MS) was performed on a fee-for-service basis on a quantitative metabolomics platform at *Biocrates Life Sciences AG*, Austria. The company had no access to phenotype information that would have permitted any data prefiltering other than objective quality control for measurement errors based on internal controls and duplicates. All metabolomics data was used as received from Biocrates. We did not apply any data correction, nor were any data points removed. The experimental metabolomics measurement technique is described in detail by patent US 2007/0004044 (accessible online at http://www.freepatentsonline.com/20070004044.html). A summary of the method can be found in [28], [49], [50]. Briefly, a targeted profiling scheme is used to quantitatively screen for known small molecule metabolites using multiple reaction monitoring, neutral loss and precursor ion scans. Quantification of the metabolites of the biological sample is achieved by reference to appropriate internal standards. The method has been proven to be in conformance with 21CFR (Code of Federal Regulations) Part 11, which implies proof of reproducibility within a given error range. It has been applied in different academic and industrial applications [51], [52]. Concentrations of all analyzed metabolites are reported in µM.

### Analyses of Metabolites

A total of 363 metabolites were targeted. Due to variability in experimental values some were excluded to ensure robustness of dataset. In the current study, 198 metabolites were used for subsequent analyses with an above 95 percent detection rate for each metabolite. Missing values were replaced with population mean for multivariate analysis.

The metabolomics dataset (for abbreviation and biochemical name see Table S1) contains 18 amino acids, eight sugars, six biogenic amines, four prostaglandins, 29 acylcarnitines, 44 sphingolipids and 89 glycerophospholipids with different head groups and are further differentiated with respect to the presence of ester (*a*) and ether (*e*) bonds in the glycerol moiety, where two letters (*aa* = diacyl, *ae* = acyl-alkyl, *ee* = dialkyl) denote that two glycerol positions are bound to a fatty acid residue, while a single letter (*a* = acyl or *e* = alkyl) indicates the presence of a single fatty acid residue. Lipid side chain composition is abbreviated as C*x:y*, where *x* denotes the number of carbons in the side chain and *y* the number of double bonds. The precise position of the double bonds and the distribution of the carbon atoms in different fatty acid side chains cannot be determined with this technology. In the current study, we used only the most likely metabolites, whereas possible alternative assignments were not indicated for cases where mapping of metabolite names to individual masses was ambiguous.

### Statistical Analysis

Pearson's correlation coefficient, hierarchical clustering methods and Euler's distance were employed and calculation was done in R platform (http://www.r-project.org/). Results of pair-wise correlations of the metabolites and clustering were illustrated by heat maps [53].

Three multivariate statistical methods, partial least squares discriminant analysis (PLS-DA), principal component analysis (PCA) and correspondence analysis (CA), were used [14], [19], [20], [21], [22]. PLS-DA used partial least squares regression models for classification and it bears some relation to PCA; Instead of finding the hyper planes of maximum variance, it finds a linear model describing some predicted variables (e.g. the behavior of smokers) in terms of observable variables (e.g. detected metabolites concentrations). PLS-DA and PCA normalizes the populations to have a mean of zero and a standard deviation of one for every metabolite; In CA normalization, however, the whole matrix is defined to be one and each element is a portion of one. CA has the advantage that the sample size needs not to be bigger than the size of variables. All the calculations were done in R platform (http://www.r-project.org/). Besides the basic packages in R, we use CA and PLS, as well as the required packages by them. PCA is using “stats” the function: princomp. All these packages can be downloaded at http://www.r-project.org.

Differences among two or more independent groups were tested by one-way ANOVA and two-tailed test [23]. Furthermore, a non-parametric Wilcoxon test was performed [24], [25] to determine whether the concentration of each small molecule was significantly different in the two groups compared.

## Supporting Information

Table S1 The abbreviation, class and biochemical name of each metabolite. For each metabolite, the abbreviation, class and biochemical name is listed in the first to third columns, respectively.
(0.05 MB XLS)

Figure S1 Classification of the 198 metabolites based on population-based KORA samples (n = 283): part 1. Each square represents the Pearson's correlation coefficient between the metabolite of the column with that of the row. Metabolite order is determined as in hierarchical clustering and the corresponding name of metabolite is shown in Figure S2, due to space limitation.
(3.42 MB TIF)

Figure S2 Classification of the 198 metabolites based on population-based KORA samples (n = 283): part 2. The corresponding name of each metabolite is shown.
(1.76 MB TIF)

Figure S3 Classification of the 198 metabolites by removing the 28 current smokers from the date set (n = 255): part 1. Each square represents the Pearson's correlation coefficient between the metabolite of the column with that of the row. Metabolite order is determined as in hierarchical clustering and the corresponding name of metabolite is shown in Figure S4, due to space limitation.
(3.47 MB TIF)

Figure S4 Classification of the 198 metabolites by removing the 28 current smokers from the date set (n = 255): part 2. The corresponding name of metabolite is shown.
(1.69 MB TIF)

Figure S5 Multivariate analysis results of 198 metabolites in 283 serum human samples. (A) Three dimensional PLS-DA results of 283 individuals. The 28 current smokers are displayed in red, while 154 former smokers and 101 non-smokers are indicated in blue and green, respectively. (B) Three dimensional PCA results. (C) Three dimensional CA results.
(1.48 MB TIF)

Figure S6 Gene expression results of AGPS (Gruber et al., 2006). Source can be found: http://www.ncbi.nlm.nih.gov/geo/gds/profileGraph.cgi?&datasetaXH3AG-CCJRMHhztsoqdPGHQLFPQLCCGG69sriID&datasetkkflcfmdegihflmmmmmlhffihfhigeeffklmmlfe&gmin35.410000&gmax292.510000&absc&gds1673&idref205401_at&annotAGPS. With kind permission of Dr. Mark Geraci.
(1.83 MB TIF)
