# Supplementary figures and images for: Metabolic Profiling Reveals Distinct Variations Linked to Nicotine Consumption in Humans — First Results from the KORA Study
Source: PLoS One. 2008 Dec 5;3(12):e3863. doi: 10.1371/journal.pone.0003863 (PMC2588343; doi:10.1371/journal.pone.0003863)

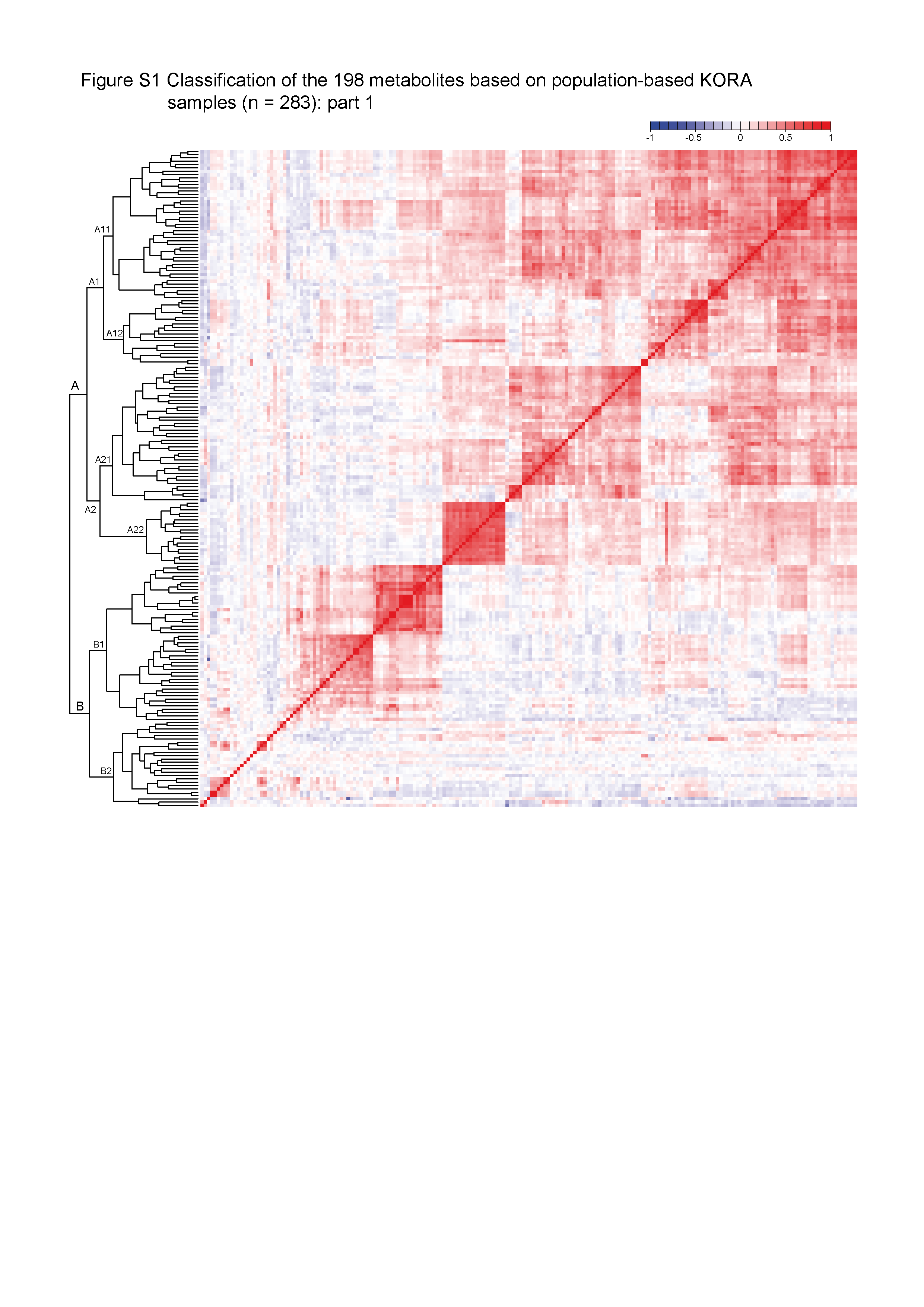

Supplement: Figure S1 — Classification of the 198 metabolites based on population-based KORA samples (n = 283): part 1. Each square represents the Pearson's correlation coefficient between the metabolite of the column with that of the row. Metabolite order is determined as in hierarchical clustering and the corresponding name of metabolite is shown in Figure S2, due to space limitation. (3.42 MB TIF) [file pone.0003863.s002.tif]

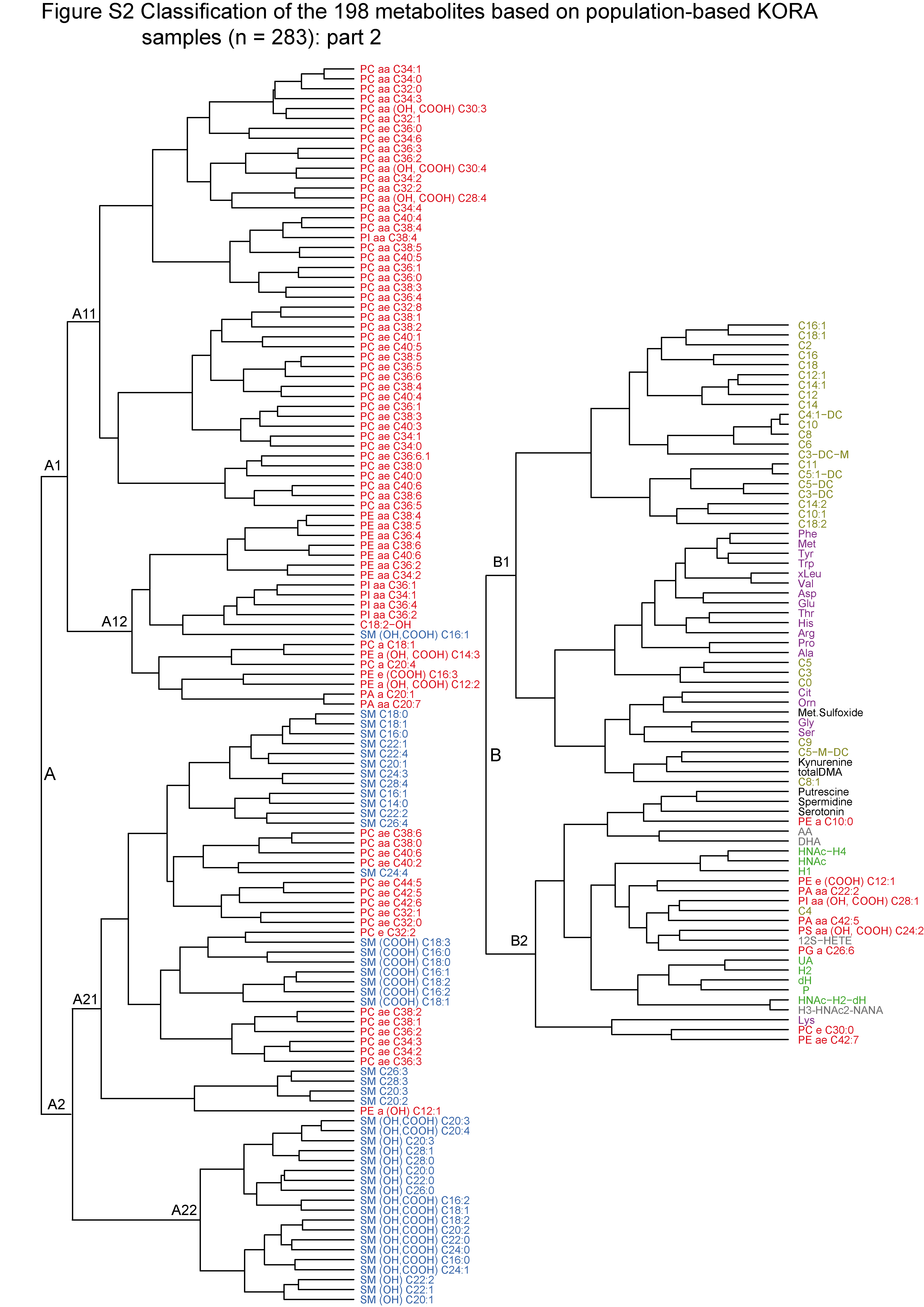

Supplement: Figure S2 — Classification of the 198 metabolites based on population-based KORA samples (n = 283): part 2. The corresponding name of each metabolite is shown. (1.76 MB TIF) [file pone.0003863.s003.tif]

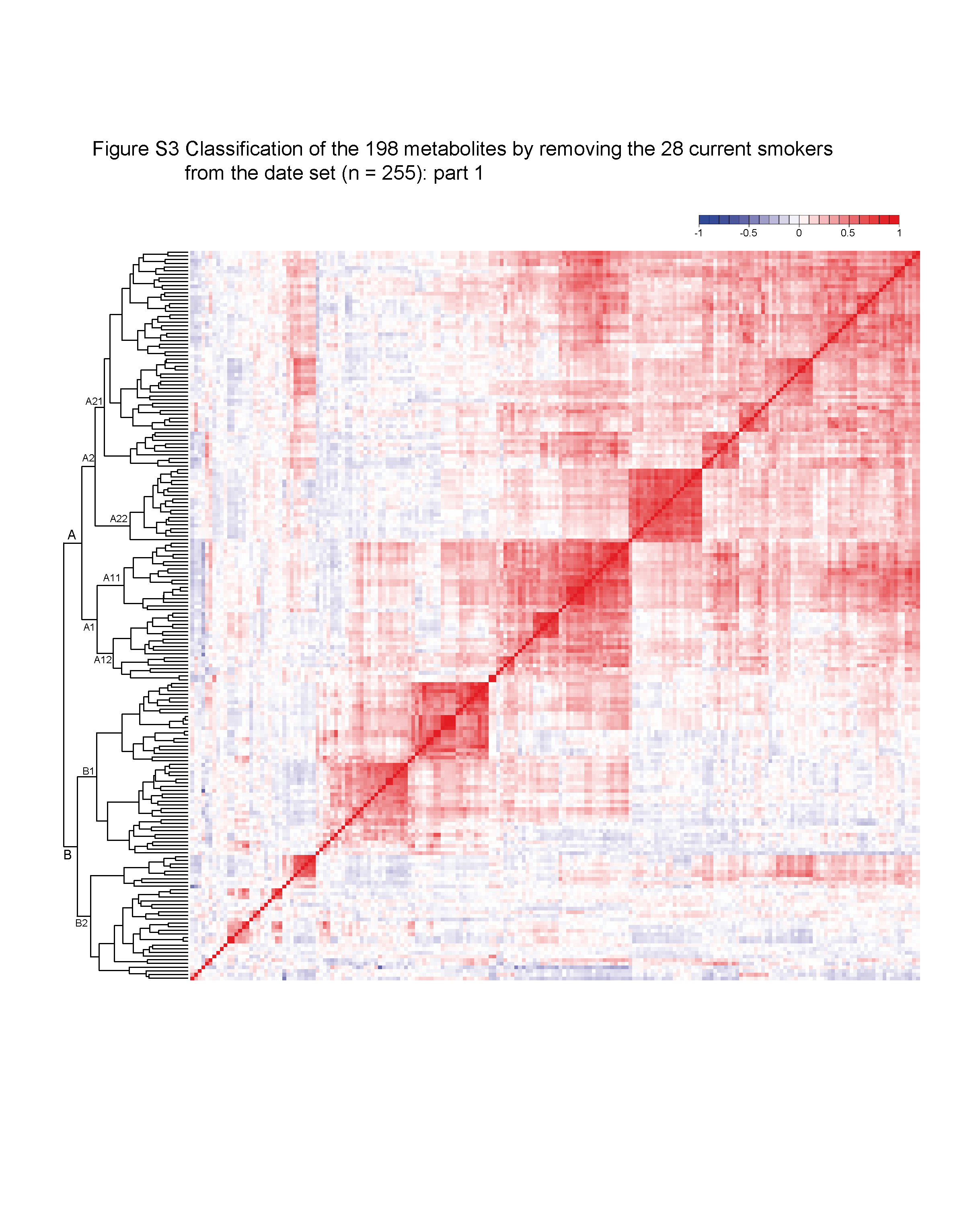

Supplement: Figure S3 — Classification of the 198 metabolites by removing the 28 current smokers from the date set (n = 255): part 1. Each square represents the Pearson's correlation coefficient between the metabolite of the column with that of the row. Metabolite order is determined as in hierarchical clustering and the corresponding name of metabolite is shown in Figure S4, due to space limitation. (3.47 MB TIF) [file pone.0003863.s004.tif]

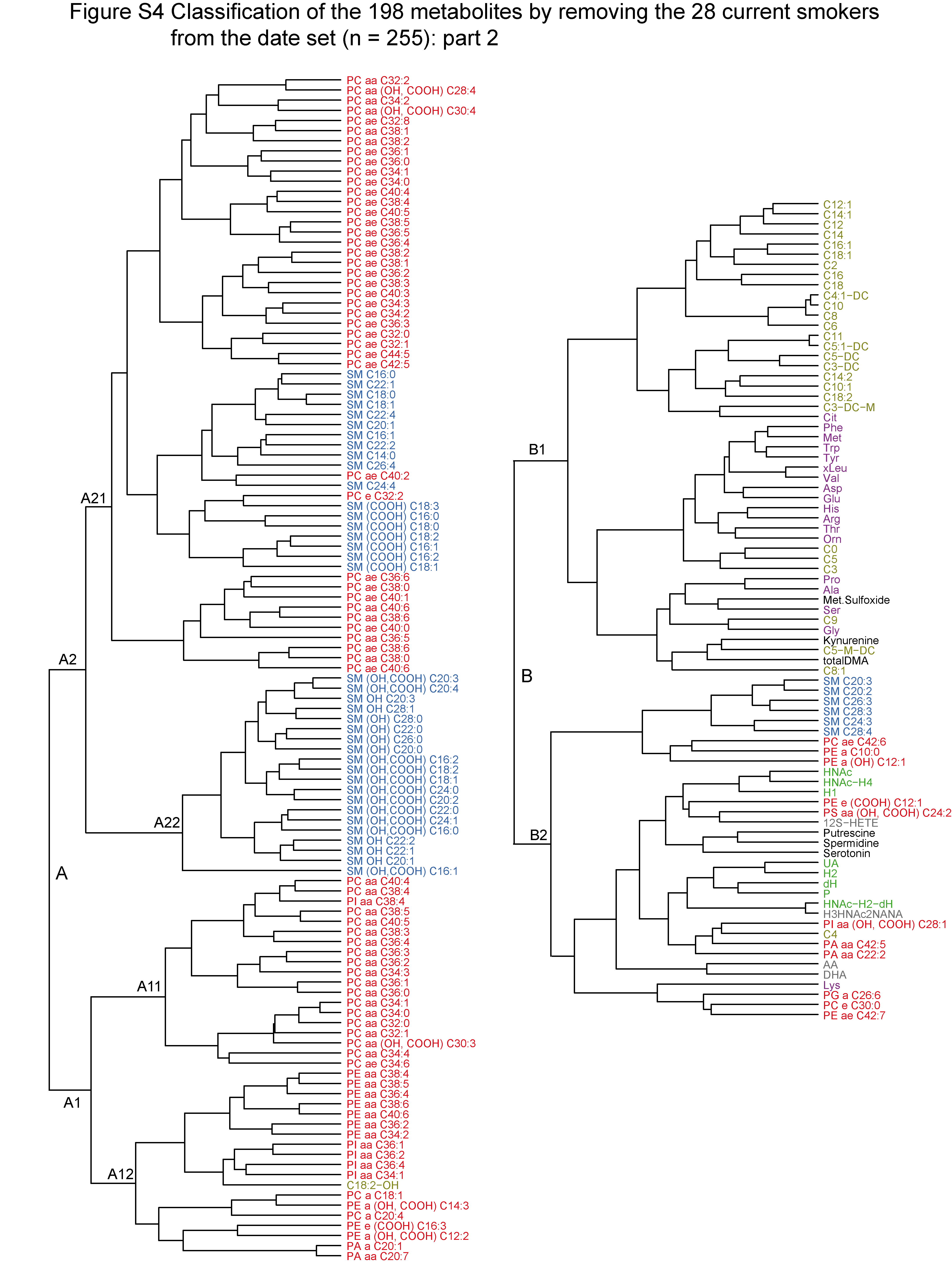

Supplement: Figure S4 — Classification of the 198 metabolites by removing the 28 current smokers from the date set (n = 255): part 2. The corresponding name of metabolite is shown. (1.69 MB TIF) [file pone.0003863.s005.tif]

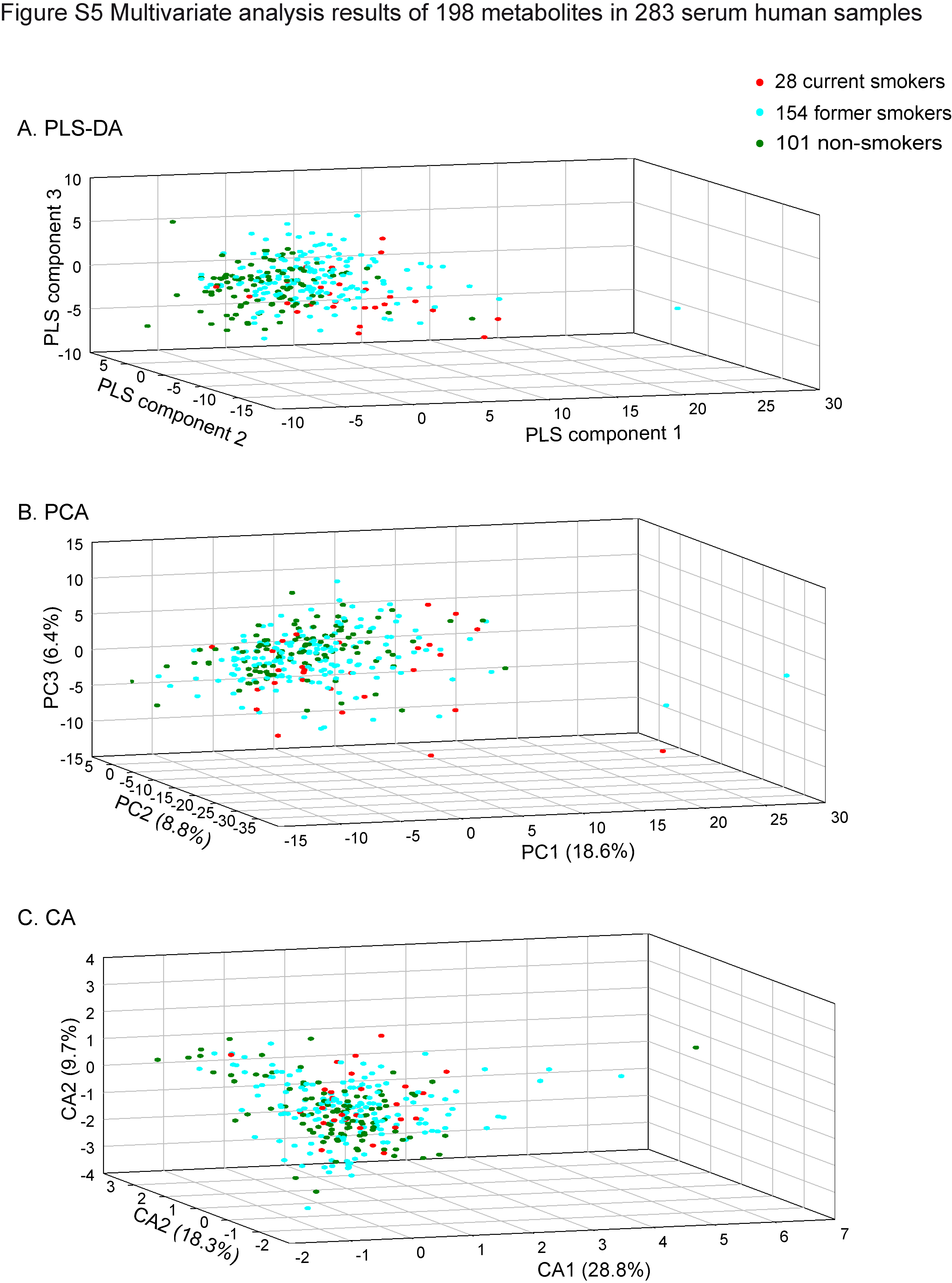

Supplement: Figure S5 — Multivariate analysis results of 198 metabolites in 283 serum human samples. (A) Three dimensional PLS-DA results of 283 individuals. The 28 current smokers are displayed in red, while 154 former smokers and 101 non-smokers are indicated in blue and green, respectively. (B) Three dimensional PCA results. (C) Three dimensional CA results. (1.48 MB TIF) [file pone.0003863.s006.tif]

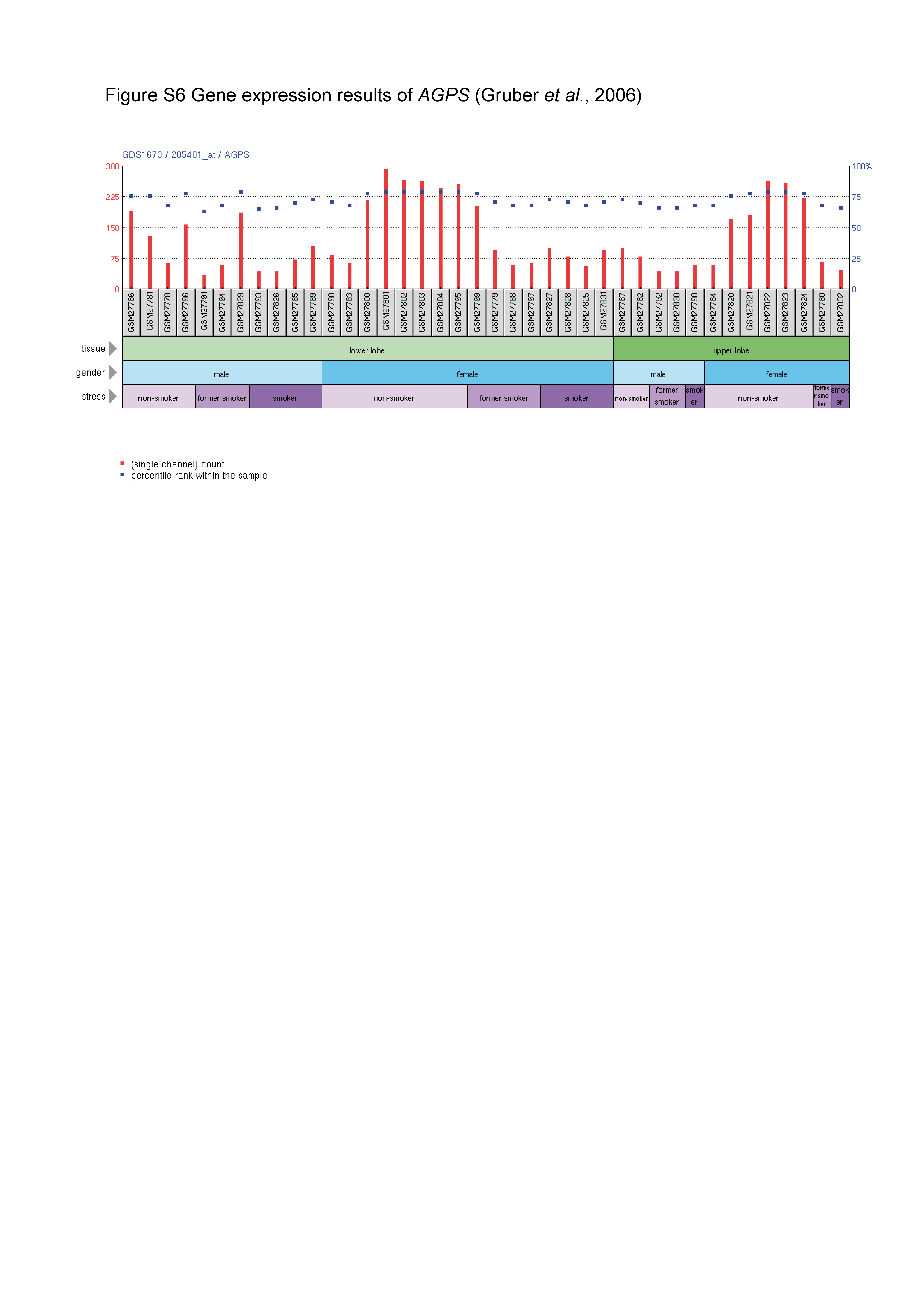

Supplement: Figure S6 — Gene expression results of AGPS (Gruber et al., 2006). Source can be found: http://www.ncbi.nlm.nih.gov/geo/gds/profileGraph.cgi?&datasetaXH3AG-CCJRMHhztsoqdPGHQLFPQLCCGG69sriID&datasetkkflcfmdegihflmmmmmlhffihfhigeeffklmmlfe&gmin35.410000&gmax292.510000&absc&gds1673&idref205401_at&annotAGPS. With kind permission of Dr. Mark Geraci. (1.83 MB TIF) [file pone.0003863.s007.tif]
